# Continuous spatio-temporal synthesis of electromagnetic fields by projected space-time Fourier transform

**DOI:** 10.1038/s44172-025-00448-9

**Published:** 2025-06-17

**Authors:** Yunlong Du, Deshuang Zhao, Chenming Guo, Junlong He, Hao Yao, Jialiang Zou, Bing-Zhong Wang

**Affiliations:** 1 School of Physics, University of Electronic Science and Technology of China, Chengdu, China; 2 Yangtze Delta Region Institute (Huzhou), University of Electronic Science and Technology of China, Huzhou, China

**Keywords:** Electrical and electronic engineering, Computational science

## Abstract

The manipulation of electromagnetic (EM) waves is important in diverse fields such as microwave sensing and wireless communication. Spatio-temporal synthesis, in particular, has attracted growing interest in recent years. Several related approaches have been explored, but their huge computational burden confines them to low-dimensional EM field synthesis. The synthesis of four-dimensional (4D) EM fields remains a challenging problem. Here, we develop the projected space-time Fourier transform (PST-FT) method to analytically control the continuous evolution of the high-dimensional EM field with time. Without iterative algorithms or pseudo-inverse matrix, it allows rapid synthesis of continuous 4D EM fields. For verification, three complicated time-varying microwave fields are synthesized, including the 4D electric field radiated by other antennas, the multi-target continuously scanning field, and the time-varying shaped uniform field. Furthermore, several corollaries and fundamental limitations are derived by integrating the developed theory and signal processing theory. Our results demonstrate the proposed theory, with potential application in reproducing EM environments, wireless communication and holographic imaging.

## Abstract (web summary)

Yunlong Du and colleagues present a general and rapid method to analytically control the continuous evolution of the electromagnetic field with time. This approach enables efficient reproduction of the four-dimensional electromagnetic environment.

## Introduction

Synthesizing a spatial EM field of the desired intensity shape within the specified region is an interesting topic due to widespread applications in various fields. As a fundamental and critical technique, the EM-field shaping can be applied to wireless communications^1–3^, wireless power transfer^4–11^, electromagnetic imaging^12–16^, electric control of spins^17–19^, hyperthermia treatment^20^, and over-the-air testing ^21^.

The classic field-shaping methods include plane-wave generation^22,23^, near-field focusing^6–8,24^, and Bessel beam generation^25^. To achieve more complex shaped EM-fields, several synthesis methods have been further developed in the past years. Since the time-reversal (TR) method was developed, it has been widely used to manipulate the EM-fields^26–28^. A TR field-shaping method was presented and successfully achieved the generation of different desired EM-fields in an enclosed aluminum cavity^29^. Subsequently, the subwavelength field shaping is realized based on the combination of TR and defective metasurfaces^30^. However, most of the developed TR synthesis methods are based on the linear superposition of multiple focused fields. Due to the incomplete orthogonality between the focused field at different positions, the TR-based field-shaping methods have high-level sidelobes. To further reduce the sidelobe levels and obtain more accurate shaped fields, many optimization methods were proposed and including phased array^31–33^, matalens antennas^34^, and metasurface^35–39^. These optimization methods provide an effective scheme to reduce the field errors and sidelobe levels, but the optimization computation is quite time-consuming due to multiple iterative numerical simulations of the spatial fields. Aiming at this limitation, a field-shaping method based on the angular spectrum projection was proposed^40^. It can analytically synthesize the microwave field of the desired intensity distribution, making the shaped-field synthesis quite fast and more accurate. However, most of the above methods only focus on the time-harmonic EM fields, which have stable spatial intensity distributions.

In recent years, there is a growing interest in synthesizing EM fields in both space and time, leading to advances in diverse applications such as reproducing the digital EM environments^41^, precise measurements^42^, wireless communication^43,44^, and coherent quantum control^45^. Although several synthesis methods have been explored, they focus only on the EM fields in three dimensions (1 temporal dimension, 2 spatial dimensions) or below, and most of them are based on pseudo-matrix technique^41^ or iterative algorithms^44^, which lead to the high computational complexity. Since the time dimension leads to a heavier computational burden, the existing synthesis methods are so time-consuming that the rapid synthesis of 4D EM field remains a challenging and unsolved problem.

In this article, we develop an analytical field synthesis method based on the projected space-time Fourier transform (PST-FT). Without a pseudo-inverse matrix and iterative algorithm, the presented method can rapidly synthesize the continuously time-varying shaped EM fields in a limited region. That is, the rapid synthesis of the 4D EM field is also allowed. Firstly, we outline the PST-FT theory based on the Hilbert space and space-time Fourier transform. According to the signal processing theory, we also derive the limitations on our approach and the array configuration. Subsequently, to validate the developed theory, we design a spherical antenna array to synthesize microwave fields with four dimensions (one temporal dimension and three spatial dimensions) and three dimensions (one temporal dimension and two spatial dimensions), respectively. The 4D electric field radiated by other antennas, the multi-target continuously scanning field, and the time-varying shaped uniform field whose shapes transition between “U”, “E”, and “S” are all efficiently synthesized.

## Methods

### Spatio-temporal expansion of 4D EM field

Consider a continuous 4D EM field $F\left(x,y,{z;t}\right)$ of duration *T* as the desired field within a limited three-dimensional (3D) synthesis region ${\Omega }_{{{{\rm{syn}}}}}$. Applying the Fourier transform^46^ in both temporal and spatial domains, we expand it as the superposition of a series of weighted single-frequency plane-wave fields:

1 $$F\left(x,y,{z;t}\right)={\sum }_{p=-\infty }^{\infty }{\sum }_{m=-\infty }^{\infty }{\sum }_{n=-\infty }^{\infty }{\sum }_{l=-\infty }^{\infty }{\widetilde{\widetilde{F}}}^{p,m,n,l}\exp ({{{\rm{j}}}}2{{{\rm{\pi }}}}{f}^{p}t)\cdot \exp (-{{{\rm{j}}}}{k}_{x}^{m}x-{{{\rm{j}}}}{k}_{y}^{n}y-{{{\rm{j}}}}{k}_{z}^{l}z)$$

where

2 $$\left\{\begin{array}{c}{f}^{p}=\frac{p}{T};{k}_{x}^{m}=\frac{2m{{{\rm{\pi }}}}}{{L}_{x}};{k}_{y}^{n}=\frac{2n{{{\rm{\pi }}}}}{{L}_{y}};{k}_{z}^{l}=\frac{2l{{{\rm{\pi }}}}}{{L}_{z}}\\ t\in \left[0,T\right]{;x}\in \left[-\frac{{L}_{x}}{2},\frac{{L}_{x}}{2}\right]{;y}\in \left[-\frac{{L}_{y}}{2},\frac{{L}_{y}}{2}\right]{;z}\in \left[-\frac{{L}_{z}}{2},\frac{{L}_{z}}{2}\right]\\ {\left(p,m,n,l\right)}^{{{{\rm{T}}}}}\in {{\mathbb{Z}}}^{4}\end{array}\right..$$

In (1), the weight ${\widetilde{\widetilde{F}}}^{p,m,n,l}$ denotes the spatio-temporal frequency spectrum of the desired field. The function $\exp ({{{\rm{j}}}}2{{{\rm{\pi }}}}{f}^{p}t)\cdot \exp (-{{{\rm{j}}}}{k}_{x}^{m}x-{{{\rm{j}}}}{k}_{y}^{n}y-{{{\rm{j}}}}{k}_{z}^{l}z)$ denotes a plane-wave field at the temporal frequency ${f}^{p}$ and the spatial frequency, i.e., the wave vector of ${{{{\boldsymbol{k}}}}}^{m,n,l}=({k}_{x}^{m},{k}_{y}^{n},{k}_{x}^{l})$. At first glance, it seems that almost all continuous 4D EM fields with complicated shapes can be linearly combined by these plane-wave fields. However, most of the plane-wave fields obtained in (1) cannot actually exist. For a plane-wave field at the temporal frequency of ${f}^{q}$, its wave vector is restricted by the dispersion relation as follows:

3 $${\left({k}_{x}^{m}\right)}^{2}+{\left({k}_{y}^{n}\right)}^{2}+{\left({k}_{z}^{l}\right)}^{2}={\left(\frac{2{{{\rm{\pi }}}}{f}^{p}}{c}\right)}^{2},$$

where *c* denotes the wave speed in the free space. To discard the evanescent waves, the wavenumbers should also satisfy the following conditions, enforcing the real-valued wave vectors:

4 $$\left\{\begin{array}{c}{\left({k}_{x}^{m}\right)}^{2}+{\left({k}_{y}^{n}\right)}^{2}\le {\left(\frac{2{{{\rm{\pi }}}}{f}^{p}}{c}\right)}^{2}\\ {\left({k}_{y}^{n}\right)}^{2}+{\left({k}_{z}^{l}\right)}^{2}\le {\left(\frac{2{{{\rm{\pi }}}}{f}^{p}}{c}\right)}^{2}\\ {\left({k}_{z}^{l}\right)}^{2}+{\left({k}_{x}^{m}\right)}^{2}\le {\left(\frac{2{{{\rm{\pi }}}}{f}^{p}}{c}\right)}^{2}\end{array}\right..$$

Thus, only the plane-wave fields satisfying both (3) and (4) are used to synthesize the 4D fields. In our work, these plane-wave fields are defined as the space-time Fourier transform primitives, whose linear superposition can construct diverse 4D EM fields.

### Theory of PST-FT

Next, we derive the space-time Fourier transform primitives from the radiated fields of a broadband antenna array and develop the PST-FT theory for 4D EM field synthesis.

**Fig. 1:**
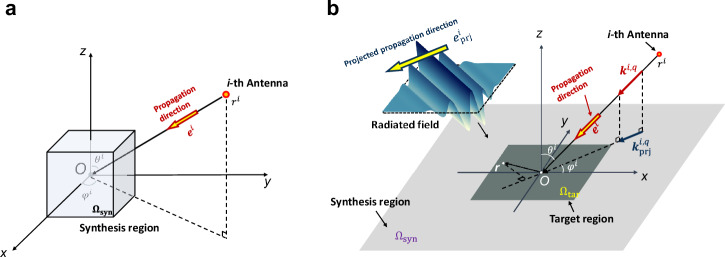
Radiation scenario of the *i*-th broadband antenna. **a** Radiation scenario with a 3D synthesis region; **b** Radiation scenario with a two-dimensional synthesis region.

Let us consider an *N*-element broadband antenna array located outside the synthesis region ${\Omega }_{{{{\rm{syn}}}}}$ in the free space, as shown in Fig. 1a. The position of the *i*-th antenna is denoted as ${{{{\boldsymbol{r}}}}}^{i}=\left({r}^{i},{\theta }^{i},{\varphi }^{i}\right),(i={{\mathrm{1,2}}},\ldots ,N)$. Assuming that the antenna is located at a distance far away from the synthesis region, the radiated field can be considered to propagate in a single direction of ${{{{\boldsymbol{e}}}}}^{i}$ within this region. Under the far-field condition for each antenna, the radiated field ${N}^{i}\left(x,y,z;t\right)$ inside the synthesis region ${\Omega }_{{{{\rm{syn}}}}}$ of the *i*-th antenna can be expressed as

5 $${N}^{i}\left(x,y,{z;t}\right)={N}^{i}\left(0,0,0{;t}\right)* \delta \left(t-\frac{{{{\boldsymbol{r}}}}\cdot {{{{\boldsymbol{e}}}}}^{i}}{c}\right),$$

where ${N}^{i}\left({{\mathrm{0,0,0}}}{;t}\right)$ denotes the radiated field arriving at the center point *O* of the synthesis region, ${{{\boldsymbol{r}}}}=x{{{{\boldsymbol{e}}}}}_{x}+y{{{{\boldsymbol{e}}}}}_{y}+z{{{{\boldsymbol{e}}}}}_{z}$ is the position vector, and ${{{{\boldsymbol{e}}}}}^{i}=-{{{{\boldsymbol{e}}}}}_{x}\sin {\theta }^{i}\cos {\varphi }^{i}-{{{{\boldsymbol{e}}}}}_{y}\sin {\theta }^{i}\sin {\varphi }^{i}-{{{{\boldsymbol{e}}}}}_{z}\cos {\theta }^{i}$ denotes the propagation direction vector of the radiated field. Considering ${S}^{i}(t)$ as the excitation signal of the *i*-th antenna, we rewrite (5) as

6 $${N}^{i}\left(x,y,{z;t}\right)={S}^{i}\left(t\right)* \frac{1}{{r}^{i}}\delta \left(t-\frac{{r}^{i}}{c}\right)* \delta \left(t-\frac{{{{\boldsymbol{r}}}}\cdot {{{{\boldsymbol{e}}}}}^{i}}{c}\right).$$

Support that the operating frequency band of the antenna array is $[{f}_{{{{\rm{L}}}}},{f}_{{{{\rm{H}}}}}]$. The corresponding temporal frequency samples are $\{{f}^{1},{f}^{2},\ldots ,{f}^{Q}\}$, where ${f}_{{{{\rm{L}}}}}\le {f}^{1} < {f}^{2} < \cdot \cdot \cdot < {f}^{Q}\le {f}_{{{{\rm{H}}}}}$. Applying the temporal Fourier transform to the right side of (6), we arrive at

7 $${N}^{i}\left(x,y,{z;t}\right)\approx {\sum }_{q=1}^{Q}{S}^{i,q}\frac{\exp \left(-{{{\rm{j}}}}{k}_{0}^{q}{r}^{i}\right)}{{r}^{i}}\exp \left(-{{{\rm{j}}}}{k}_{x}^{i,q}x-{{{\rm{j}}}}{k}_{y}^{i,q}y-{{{\rm{j}}}}{k}_{z}^{i,q}z\right)\exp \left({{{\rm{j}}}}2{{{\rm{\pi }}}}{f}^{q}t\right),$$

where

$$\left\{\begin{array}{c}{k}_{0}^{q}=\frac{2{{{\rm{\pi }}}}{f}^{q}}{c}\\ {k}_{x}^{i,q}=-{k}_{0}^{q}\sin {\theta }^{i}\cos {\varphi }^{i}\\ {k}_{y}^{i,q}=-{k}_{0}^{q}\sin {\theta }^{i}\sin {\varphi }^{i}\\ {k}_{z}^{i,q}=-{k}_{0}^{q}\cos {\theta }^{i}\end{array}\right..(8)$$

In (7), ${S}^{i,q}$ denotes the temporal frequency spectrum of ${S}^{i}(t)$ at the frequency of ${f}^{q}$. Equation (7) implies that the radiated field of the *i*-th antenna can be decomposed into *Q* primitives, expressed as

9 $${N}^{i}\left(x,y,{z;t}\right)\approx {\sum }_{q=1}^{Q}{S}^{i,q}\frac{\exp \left(-{{{\rm{j}}}}{k}_{0}^{q}{r}^{i}\right)}{{r}^{i}}{B}^{i,q}\left(x,y,{z;t}\right)$$

with the space-time Fourier transform primitives

10 $${B}^{i,q}\left(x,y,{z;t}\right)=\exp \left(-{{{\rm{j}}}}{k}_{x}^{i,q}x-{{{\rm{j}}}}{k}_{y}^{i,q}y-{{{\rm{j}}}}{k}_{z}^{i,q}z\right)\exp \left({{{\rm{j}}}}2{{{\rm{\pi }}}}{f}^{q}t\right)$$

Accordingly, we can obtain the set of primitives corresponding to the array as

11 $$\left\{{B}^{i,q}\left(x,y,{z;t}\right),i\in \left\{1,2,\ldots ,N\right\},q\in \left\{1,2,\ldots ,Q\right\}\right\}$$

These primitives span the synthesis subspace ${{{\mathcal{S}}}}$ of the array:

12 $${{{\mathcal{S}}}}=\left\{u\left|u={\sum }_{i=1}^{N}{\sum }_{q=1}^{Q}{w}^{i,q}{B}^{i,q}(x,y,{z;t})\right.\right\}$$

where ${w}^{i,q}{\mathbb{\in }}{\mathbb{C}}$. Thus, for a 4D desired field $F(x,y,{z;t})$, the optimal synthesized field ${F}_{{{{\rm{syn}}}}}(x,y,{z;t})$ can be calculated by the projection as

13 $${F}_{{{{\rm{syn}}}}}(x,y,{z;t})={{{{\bf{P}}}}}_{{{{\rm{syn}}}}}\left(F\right)={\sum }_{i=1}^{N}{\sum }_{q=1}^{Q}{v}^{i,q}{B}^{i,q}\left(x,y,{z;t}\right),$$

where

14 $${v}^{i,q}=\frac{\left\langle F,{B}^{i,q}\right\rangle }{\left\langle {B}^{i,q},{B}^{i,q}\right\rangle }=\frac{\int\limits_{0}^{T}{\iiint }_{{\Omega }_{{{{\rm{syn}}}}}}F\left(x,y,{z;t}\right){\left({B}^{i,q}\left(x,y,{z;t}\right)\right)}^{* }{{{\rm{d}}}}x{{{\rm{d}}}}y{{{\rm{d}}}}z{{{\rm{d}}}}t}{\left\langle {B}^{i,q}\left(x,y,{z;t}\right),{B}^{i,q}\left(x,y,{z;t}\right)\right\rangle }.$$

In (13), ${{{{\bf{P}}}}}_{{{{\rm{syn}}}}}$ denotes the projection operator to the synthesis subspace ${{{\mathcal{S}}}}$, and and ${v}^{i,q}$ can be computed efficiently with the use of FT.

Combining (14) and (9), the spectrum of the excitation signal is calculated by

15 $${S}^{i,q}={r}^{i}\exp \left({{{\rm{j}}}}{k}_{0}^{q}{r}^{i}\right){v}^{i,q}.$$

Finally, applying the temporal Fourier transform, we obtain the excitation signal of each antenna as

16 $${S}^{i}\left(t\right){\mathfrak{=}}{\mathfrak{R}}\left[{\sum }_{q=1}^{Q}{S}^{i,q}\exp \left({{{\rm{j}}}}2{{{\rm{\pi }}}}{f}^{q}t\right)\right]$$

In addition, it is worth mentioning that the PST-FT theory can also be used to synthesize time-varying fields in a two-dimensional (2D) synthesis region. Figure 1b shows the radiation scenario with the 2D synthesis region. Similar to the case of the 3D synthesis region, assuming that the antenna is far away from the synthesis region, the radiated field can also be considered to propagate in a single direction within this region. This means that we can still decompose it into a linear superposition of a set of primitives. Thus, the array excitation signals can be calculated by the projection similar to (13). The detailed derivation for the case of a 2D synthesis region is shown in Supplementary Note 1.

## Results

### Design of a spherical antenna array

**Fig. 2:**
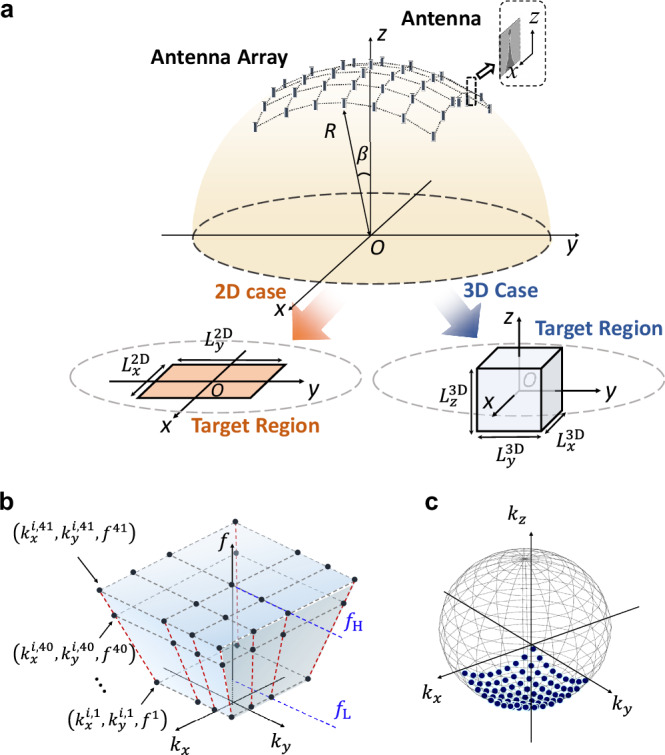
Diagram and the spatio-temporal frequency samples of the spherical antenna array. **a** Diagram of the designed antenna array and the target region. **b** The distribution of the spatio-temporal frequency samples corresponding to the array in the 2D case. **c** In the 3D case, the distribution of the spatial frequency samples corresponding to the array at a single temporal frequency.

Figure 2a presents the structure of the spherical antenna array we designed for synthesizing the time-varying shaped fields. The operating frequency band $\left[{f}_{{{{\rm{L}}}}},{f}_{{{{\rm{H}}}}}\right]$ of each antenna is 1–2 GHz, from which 41 frequencies are selected evenly as the frequency samples $\left\{{f}^{1},{f}^{2},\ldots ,{f}^{41}\right\}$. For equal radiation field amplitudes within the target region, the array is composed of 81 Vivaldi antennas distributed on the surface of a half-sphere with radius $R=11{\lambda }_{{{{\rm{L}}}}}=3.3{{{\rm{m}}}}$ and the angle $\beta =30^\circ$, where ${\lambda }_{{{{\rm{L}}}}}=2{{{\rm{\pi }}}}{f}_{{{{\rm{L}}}}}/c$. To sample uniformly in the spatial frequency domain, these antennas are placed at equal *x*-interval and equal *y*-interval (see Supplementary Note 2 for detailed derivation and geometries). To ensure that the radiation fields within the target region have the same polarization direction, all antennas are parallel to the *xoz*-plane with their surface currents polarized at the *x*-direction. For the time-varying synthesis in the 2D and 3D target region, as shown in Fig. 1a, the target region sizes are set to ${L}_{x}^{2{{{\rm{D}}}}}\times {L}_{y}^{2{{{\rm{D}}}}}=1.6\times 1.6{{{{\rm{m}}}}}^{2}$ and ${L}_{x}^{3{{{\rm{D}}}}}\times {L}_{y}^{3{{{\rm{D}}}}}\times {L}_{z}^{3{{{\rm{D}}}}}=1.2\times 1.2\times 1.2{{{{\rm{mm}}}}}^{3}$, respectively. In the 2D case, the spatio-temporal frequency samples corresponding to the array are depicted in Fig. 2b. ${k}_{x}$ and ${k}_{y}$ denote the spatial frequencies in the *x*- and *y*-directions, respectively. Each red dashed line denotes the spatio-temporal frequency spectrum corresponding to each antenna, and the black dots represent the spatio-temporal frequency samples we applied in the simulations. In the 3D case, the spatial frequency samples at a single temporal frequency are shown in Fig. 2c. ${k}_{z}$ denotes the spatial frequency in the *z*-direction.

### Synthesis of time-varying shaped fields

The PST-FT theory is validated by full-wave simulations at microwave frequencies. In the simulations, three complex desired fields are synthesized, one in the 3D synthesis region and two in the 2D synthesis region.

**Fig. 3:**
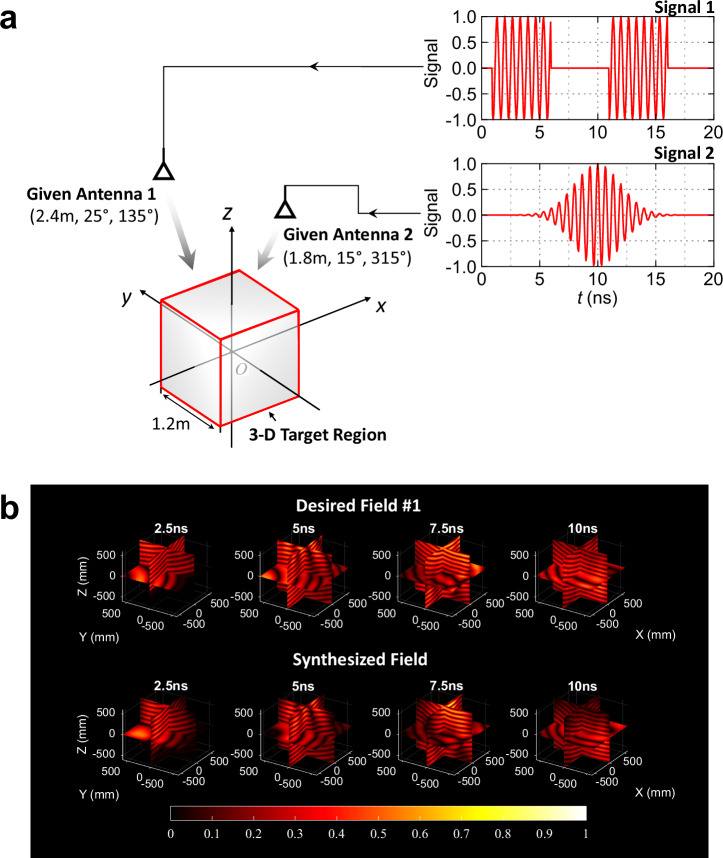
Reproduction of the 4D radiated electric field. **a** Schematic of the simulation obtaining the desired field #1; **b** Normalized intensity distribution of the electric fields at different moments (2.5, 5, 7.5, and 10 ns), where the first row presents the slices of the desired field #1 and the second row presents the slices of the field synthesized by the spherical antenna array.

The first one is a 4D electric field radiated by other antennas. These given antennas are excited simultaneously by different signals, as shown in Fig. 3a and listed in Supplementary Data 1. Their radiation field within the 3D target region of $1.2{{{\rm{m}}}}\times 1.2{{{\rm{m}}}}\times 1.2{{{\rm{m}}}}$ is set as the desired field #1, whose duration is 10 ns and slices are shown in the first row of Fig. 3b. The task is to reproduce this radiation field in the 3D target region by the spherical array. Based on PST-FT theory, we spent 49 s to obtain the excitation signals of the spherical array, as listed in Supplementary Data 2. The simulation results of the synthesized field are presented in the second row of Fig. 3b. All fields are normalized with respect to the maximum value over the full duration. The simulation results illustrate a good match between the desired field and synthesized field (also see Supplementary Movie 1), with a mean square error (MSE) of 3% (see Supplementary Note 3 for detailed calculation). The calculation scale and computer information are available in Supplementary Note 4.

**Fig. 4:**
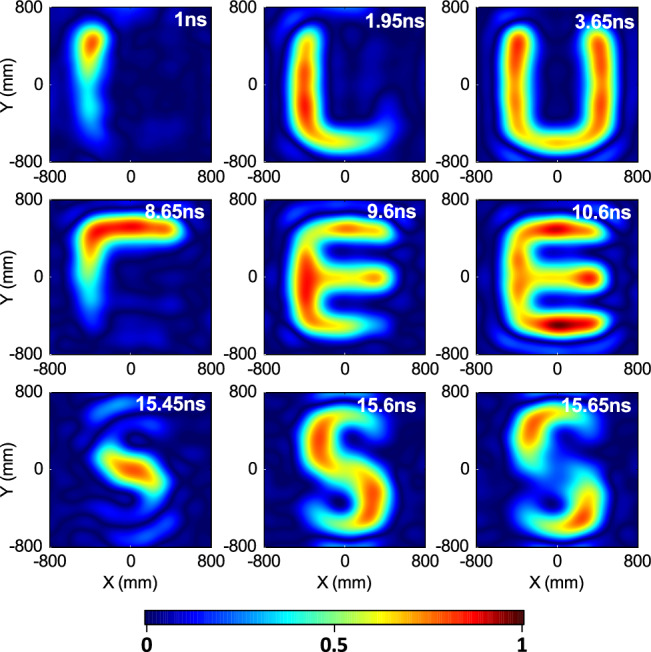
Intensity evolution of the synthesized time-varying shaped uniform field.

**Fig. 5:**
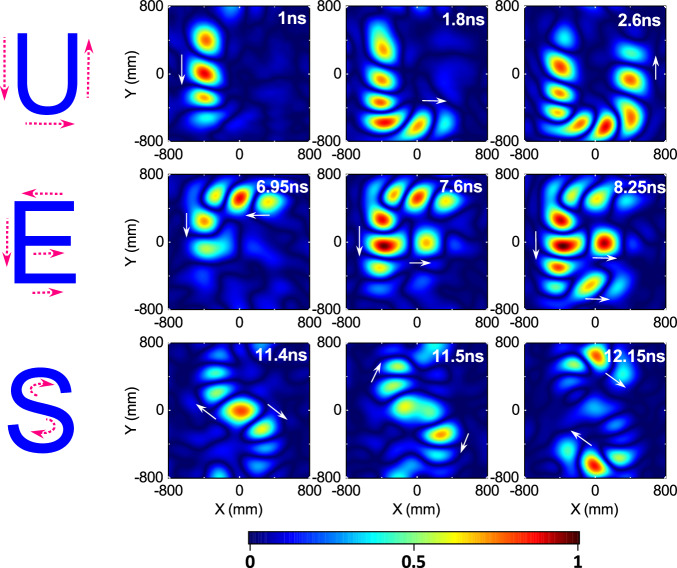
Intensity evolution of the synthesized multi-target continuously scanning field. The continuous scanning trajectory is illustrated in the leftmost column, which dynamically transitions through “U”-, “E”-, and “S”-shapes.

The desired field #2 is a time-varying shaped uniform field within the 2D target region, with the duration of 20 ns and the shape transitions between “U”, “E”, and “S”. The corresponding synthesized field at some moments are presented in Fig. 4. The animations and excitation signals are available in the Supplementary Movie 2 and Supplementary Data 3, respectively. The successful generation of the animated transitions between the “U”-, “E”-, and “S”-shapes can be observed. The desired field #3 is a multi-target continuously scanning field within the 2D target region. Its duration is 20 ns and the trajectory is “U”-, “E”-, and “S”-shaped. The corresponding synthesized field at some moments are presented in Fig. 5. The animations and excitation signals are available in the Supplementary Movie 3 and Supplementary Data 4, respectively. It shows the superior scanning focus, either along the straight trajectory or along the curved trajectory.

## Discussion and conclusion

The presented PST-FT theory allows the rapid synthesis of larger-scale arrays and fields, with the growth rate of calculation time much lower than that of the number of antennas and field sampling points (for details see Supplementary Note 4). Integrating the PST-FT theory and signal processing theory, some limitations on the synthesized field can be derived. First, to avoid the aliasing distortion in the synthesized field, the size of the desired field in the *x*- and *y*-directions cannot exceed 2400 mm, and the duration must be limited to 40 ns. More intensive array and temporal frequency sampling can overcome these limitations (see Supplementary Note 5 for detailed derivation). Second, the sidelobes between adjacent focal points are attributed to the ringing effect, restricted by the bandwidth of the antenna. Combining smooth reference mask (SRM)^33^ with the presented PST-FT theory, we can depress the sidelobe levels of the time-varying synthesized fields (see Supplementary Note 6 for verification). In addition, it should be noted that the presented theory only solves the synthesis problem of the single polarization component. With the antenna far away from the synthesis region, we neglect the polarization components orthogonal to the polarization direction of the excited antenna, which have been confirmed to be relatively weak by full-wave simulations (see Supplementary Note 7 for details).

In conclusion, we present a general theory for synthesizing the shaped EM field, which is continuously time-varying. Different from the previous computationally intensive approaches, the proposed PST-FT theory allows rapid synthesis of 4D EM fields. Several intriguing 3D and 4D EM fields are efficiently synthesized, demonstrating application potential in radar testing, wireless communication, holographic imaging and antenna measurement. Since the developed method only controls the polarization component consistent with the excited antenna, it has the potential to combine multi-polarized antennas with the polarization decomposition technique to synthesize different components of the vector field separately. Furthermore, although we have only validated for the microwave fields, the proposed theory could be extended to other frequency ranges, such as the acoustic waves and millimeter waves.

## Supplementary information

Supplementary Information

Description of Additional Supplementary Files

Supplementary Movie 1

Supplementary Movie 2

Supplementary Movie 3

Supplementary Data 1

Supplementary Data 2

Supplementary Data 3

Supplementary Data 4

## Data Availability

All relevant data are available in the paper and the Supplement Information Files, or from the authors upon reasonable request.

## Code availability

The codes implementing the PST-FT theory are available from the corresponding authors upon reasonable request.
