# Supplementary material for: Continuous spatio-temporal synthesis of electromagnetic fields by projected space-time Fourier transform
Source: Commun Eng. 2025 Jun 17;4:110. doi: 10.1038/s44172-025-00448-9 (PMC12174354; doi:10.1038/s44172-025-00448-9)
Supplement: Supplementary file 1 — Supplementary Information [file 44172_2025_448_MOESM1_ESM.pdf]

Supplementary Information for

Continuous Spatio-Temporal Synthesis of Electromagnetic Fields by Projected Space-Time Fourier Transform

by Du et al.

Supplementary Note 1: Theory of projected space-time Fourier transform synthesis in the two-dimensional synthesis region

Based on the projected space-time Fourier transform (PST-FT) theory presented in the main text, we further extend it to the case of two-dimensional (2D) synthesis region. The detailed derivation is given in this supplementary note.

Consider a time-varying shaped microwave field as the desired field $F(x, y; t)$, which is located in a limited 2D synthesis region Ω_{syn} and duration is T. Based on the theory of Fourier transform in the time domain, we can first expand it as the sum of a series of weighted time-harmonic fields with discrete frequencies

$$F(x, y; t) = \sum_{p=-\infty}^{\infty} \tilde{F}^{p}(x, y) \exp(\mathrm{j}2\pi f^{p} t), \tag{1}$$

where

$$\begin{cases} f^{p} = \dfrac{p}{T}, p \in \mathbb{Z} \\ x \in \left[-\dfrac{L_x}{2}, \dfrac{L_x}{2}\right]; y \in \left[-\dfrac{L_y}{2}, \dfrac{L_y}{2}\right]; t \in [0, T] \end{cases}. \tag{2}$$

The weight $\tilde{F}^{p}(x, y)$ represents the temporal-frequency spectrum of $F(x, y; t)$ at the frequency of f^{p} and the position of (x, y). L_x and L_y denote the length of the synthesis region Ω_{syn} along x- and y-directions respectively, and T denotes the duration of the desired field $F(x, y; t)$.

Furthermore, the weight $\tilde{F}^{p}(x, y)$ can be expanded with the sum of a series of spatial-frequency spectra via the two-dimensional Fourier transform in the space domain as

$$\tilde{F}^{p}(x, y) = \sum_{m=-\infty}^{\infty} \sum_{n=-\infty}^{\infty} \tilde{\tilde{F}}^{p,m,n} \exp\left(-\mathrm{j}k_x^m x - \mathrm{j}k_y^n y\right) \tag{4}$$

where

$$\begin{cases} k_x^m = \dfrac{2m\pi}{L_x}; k_y^n = \dfrac{2n\pi}{L_y} \\ (m,n)^{\mathrm{T}} \in \mathbb{Z}^2 \end{cases}. \tag{5}$$

$k_x^m$and k_y^n denote the spatial frequencies in the *x*- and *y*-directions, respectively. In the field of electromagnetics, they also represent the *x*- and *y*-components of the wave vector. With the above twice Fourier transforms, we arrive at

$$F(x,y;t) = \sum_{p=-\infty}^{\infty} \sum_{m=-\infty}^{\infty} \sum_{n=-\infty}^{\infty} \left(\tilde{\tilde{F}}^{p,m,n} \exp(\mathrm{j}2\pi f^p t) \cdot \exp\left(-\mathrm{j}k_x^m x - \mathrm{j}k_y^m y\right) \right). \tag{6}$$

Generally, Supplementary Equation (6) is called the space-time Fourier transform (ST-FT) of $F(x,y;t)$. Here, we denote the amplitude $\tilde{\tilde{F}}^{p,m,n}$ as the spatio-temporal frequency spectrum of $F(x,y;t)$, and the function $\exp\left(-\mathrm{j}k_x^m x - \mathrm{j}k_y^n y\right)\exp(\mathrm{j}2\pi f^p t)$ as a plane-wave field propagating in the *xoy*-plane at the temporal frequency of f^p and the spatial frequency, i.e., the wave vector of $\boldsymbol{k}^{m,n} = \left(k_x^m, k_y^n\right)$. The equation (6) implies that almost any time-varying shaped microwave field can be decomposed into the sum of a set of weighted plane-wave fields at different spatio-temporal frequencies.

In our study, we define the plane-wave fields $\exp\left(-\mathrm{j}k_x^m x - \mathrm{j}k_y^n y\right)\exp(\mathrm{j}2\pi f^p t)$ as the space-time Fourier transform primitives, i.e., the basis functions to compose the desired time-varying spatially-shaped microwave field. These primitives can be denoted as a set by

$$\{B^{p,m,n}(x,y;t), (p,m,n)^{\mathrm{T}} \in \mathbb{Z}^3\} \tag{7}$$

where

$$B^{p,m,n}(x,y;t) = \exp\left(-\mathrm{j}k_x^m x - \mathrm{j}k_y^n y\right)\exp(\mathrm{j}2\pi f^p t). \tag{8}$$

Mathematically, all the possible fields expanded with these primitives can be grouped into a field space $\mathcal{H}$ as follows

$$\mathcal{H} = \left\{ u \middle| \sum_{p=-\infty}^{\infty} \sum_{m=-\infty}^{\infty} \sum_{n=-\infty}^{\infty} c^{p,m,n} B^{p,m,n}(x,y;t) \right\} \tag{9}$$

where $c^{p,m,n} \in \mathbb{C}$. For the desired field $F(x,y;t)$, the expansion coefficients $c^{p,m,n}$ correspond to its spatial-temporal frequency spectra $\tilde{\tilde{F}}^{p,m,n}$, which can be calculated with the projection of the desired field onto these primitives, as follows:

$$c^{p,m,n} = \tilde{\tilde{F}}^{p,m,n} = \mathbf{P}^{p,m,n}(F) = \frac{\langle F(x,y;t), B^{p,m,n}(x,y;t)\rangle}{\langle B^{p,m,n}(x,y;t), B^{p,m,n}(x,y;t)\rangle} \tag{10}$$

where

$$\langle F(x,y;t), B^{p,m,n}(x,y;t)\rangle = \int_{0}^{T} \int_{-L_y}^{L_y} \int_{-L_x}^{L_x} F(x,y;t) \cdot \left(B^{p,m,n}(x,y;t)\right)^{*} \mathrm{d}x\mathrm{d}y\mathrm{d}t. \tag{11}$$

$\mathbf{P}^{p,m,n}(\cdot)$ denotes the projection operator corresponding to the primitive $B^{p,m,n}(x,y,t)$. $\tilde{\tilde{F}}^{p,m,n}$ can be calculated with the inner product between $F(x,y;t)$ and each primitive.

Then, we derive the primitives from the radiated fields by broadband antenna array and obtain the synthesis subspace corresponding to the array. Let us consider an *N*-element broadband antenna array located outside the synthesis region Ω_{sy} in the free space with the position $\boldsymbol{r}^i = \left(r^i, \theta^i, \varphi^i\right), (i = 1,2,\ldots,N)$ of each antenna, as shown in Fig. 1b. In the synthesis region Ω_{syn}, the radiated field of the *i*-th antenna can be considered to propagate in a single direction of $\boldsymbol{e}^i$ by assuming that the antenna is located at a distance far away from this region. Under the far-field

condition, the radiated field $N^i(x,y;t)$ inside the synthesis region Ω_{syn} of the i-th antenna can be represented as

$$N^i(x,y;t) = N^i(0,0;t) * \delta\left(t - \frac{\boldsymbol{r}\cdot\boldsymbol{e}^i}{c}\right) \tag{12}$$

where

$$\begin{cases} \boldsymbol{r} = x\boldsymbol{e}_x + y\boldsymbol{e}_y \\ \boldsymbol{e}^i = -\boldsymbol{e}_x \sin\theta^i \cos\varphi^i - \boldsymbol{e}_y \sin\theta^i \sin\varphi^i - \boldsymbol{e}_z \cos\theta^i \end{cases}. \tag{13}$$

$N^i(0,0;t)$ denotes the radiated field arriving at the center point O of the synthesis region Ω_{syn}. $\delta(\cdot)$ is the impulse function and c is the wave speed. The operator $*$ donates the convolution operation, $\boldsymbol{r}$ represents the position vector and $\boldsymbol{e}^i$ denotes the propagation direction of the radiated field. Considering $S^i(t)$ as the excitation signal of the i-th antenna, then $N^i(0,0;t)$ can be calculated as

$$N^i(0,0;t) = S^i(t) * \frac{1}{r^i}\delta\left(t - \frac{r^i}{c}\right). \tag{14}$$

Then the radiated field $N^i(x,y;t)$ of the i-th antenna can be expressed as

$$N^i(x,y;t) = S^i(t) * \frac{1}{r^i}\delta\left(t - \frac{r^i}{c}\right) * \delta\left(t - \frac{\boldsymbol{r}\cdot\boldsymbol{e}^i}{c}\right). \tag{15}$$

Support that the operating frequency band of the antenna array is $[f_{\mathrm{L}}, f_{\mathrm{H}}]$. From the set of frequencies $\{f^p\}$ described by Supplementary Equation (3), some frequencies included in the operating frequency band are selected as the frequency samples $\{f^1, f^2, \dots, f^Q\}$ of the spectrum of the antenna array, where $f_{\mathrm{L}} \le f^1 < f^2 <\dots< f^Q \le f_{\mathrm{H}}$. Applying the Fourier transform in the time domain to the right side of (15), we get

$$N^i(x,y;t) \approx \sum_{q=1}^{Q} S^{i,q} \frac{\exp\left(-\mathrm{j}k_0^q r^i\right)}{r^i} \exp\left(-\mathrm{j}\boldsymbol{k}_{\mathrm{prj}}^{i,q} \cdot \boldsymbol{r}\right) \exp(\mathrm{j}2\pi f^q t) \tag{16}$$

where

$$\begin{cases} k_0^q = 2\pi f^q / c \\ \boldsymbol{k}_{\mathrm{prj}}^{i,q} = -\boldsymbol{e}_x k_0^q \sin\theta^i \cos\varphi^i - \boldsymbol{e}_y k_0^q \sin\theta^i \sin\varphi^i \end{cases}. \tag{17}$$

$S^{i,q}$ denotes the temporal-frequency spectrum of $S^i(t)$ at the frequency of f^q, $k_0^q = 2\pi f^q/c$ is the free-space wavenumber, and $\boldsymbol{k}_{\mathrm{prj}}^{i,q}$ represents the projection of the wave vector $\boldsymbol{k}^{i,q}$ onto the *xoy*-plane, as shown in Fig. 1. It is evident that the function $\exp\left(-\mathrm{j}\boldsymbol{k}_{\mathrm{prj}}^{i,q} \cdot \boldsymbol{r}\right)\exp(\mathrm{j}2\pi f^q t)$ can be regarded as one of the primitives described by Supplementary Equation (8). Thus, Supplementary Equation (16) illustrates that the radiated field $N^i(x,y;t)$ within Ω_{syn} of the *i*-th antenna can be decomposed into a set of primitives, which can be expressed as

$$\left\{B^{i,q}(x,y;t), q \in \{1,2,\dots,Q\}\right\} \tag{18}$$

where

$$\begin{cases} B^{i,q}(x,y;t) = \exp\left(-\mathrm{j}k_x^{i,q} x - \mathrm{j}k_y^{i,q} y\right)\exp(\mathrm{j}2\pi f^q t) \\ k_x^{i,q} = -k_0^q \sin\theta^i \cos\varphi^i \\ k_y^{i,q} = -k_0^q \sin\theta^i \sin\varphi^i \end{cases}. \tag{19}$$

In Supplementary Equation (19), f^q denotes the temporal frequency of each primitive, $k_x^{i,q}$ and $k_y^{i,q}$ denote the spatial frequency in the *x*- and *y*-directions of each primitive respectively. Subsequently, the set of primitives corresponding to the antenna array can be obtained as

$$\left\{B^{i,q}(x,y;t), i \in \{1,2,\dots,N\}, q \in \{1,2,\dots,Q\}\right\}. \tag{20}$$

Then the radiated field $F_{\mathrm{syn}}(x,y;t)$ within the synthesis region Ω_{syn} of the whole antenna array can be expressed as the sum of these primitives:

$$F_{\mathrm{syn}}(x,y;t)=\sum_{i=1}^{N}N^{i}(x,y;t)\approx\sum_{i=1}^{N}\sum_{q=1}^{Q}w^{i,q}B^{i,q}(x,y;t) \tag{21}$$

where the weight $w^{i,q}$ can be calculated by Supplementary Equation (16) as

$$w^{i,q}=S^{i,q}\frac{\exp\left(-\mathrm{j}k_0^{q}r^{i}\right)}{r^{i}}. \tag{22}$$

It illustrates that the weight $w^{i,q}$ of each primitive in (21) can be arbitrarily valued by the spectrum $S^{i,q}$ of the excitation signal $S^{i}(t)$. Therefore, the radiated field F_{syn} of the antenna array can be grouped into the field space $\mathcal{S}$ as follows

$$F_{\mathrm{syn}}(x,y;t)\in\mathcal{S}=\left\{u\,\middle|\,u=\sum_{i=1}^{N}\sum_{q=1}^{Q}w^{i,q}B^{i,q}(x,y;t)\right\}. \tag{23}$$

Since $\mathcal{S}$ is a subspace of $\mathcal{H}$ described by Supplementary Equation (9) and contains all the possible fields synthesized by the antenna array, it is defined as the synthesis subspace of this array. For a field synthesis problem, we need to find the optimal field within the synthesis subspace $\mathcal{S}$.

Subsequently, we derive the optimal synthesized field and the corresponding excitation signals of the antenna array. In most cases, the desired field $F(x,y;t)$ has the following form

$$F(x,y;t)=\begin{cases}F_{\mathrm{tar}}(x,y;t), (x,y)\in\Omega_{\mathrm{tar}}, t\in[0,T_{\mathrm{tar}}]\\ 0, \qquad (x,y)\notin\Omega_{\mathrm{tar}}, t\in(T_{\mathrm{tar}},T]\end{cases} \tag{24}$$

where $F_{\mathrm{tar}}(x,y;t)$ denotes the desired field within the target region, $\Omega_{\mathrm{tar}}\subset\Omega_{\mathrm{syn}}$ denotes the target region we focus on, as shown in Fig. 1b in the main text, and $T_{\mathrm{tar}}<T$. In general, the field synthesis problem can be formulated into a linear least-squares problem:

$$\min_{(S^{1,1},\dots,S^{N,Q})^{\mathrm{T}}} \left\| F_{\mathrm{syn}} - F \right\|^2 \tag{25}$$

where $\left\| F_{\mathrm{syn}} - F \right\|^2 = \langle F_{\mathrm{syn}} - F, F_{\mathrm{syn}} - F \rangle$. Since the synthesized field $F_{\mathrm{syn}} \in \mathcal{S}$, the optimal solution will be obtained when $F_{\mathrm{syn}}(x, y; t)$ is equal to the projection of $F(x, y; t)$ onto the synthesis subspace $\mathcal{S}$. This projection F_{prj} can be calculated as

$$F_{\mathrm{prj}}(x, y; t) = \mathbf{P}_{\mathrm{syn}}(F) = \sum_{i=1}^{N} \sum_{q=1}^{Q} \mathbf{P}^{i,q}(F) = \sum_{i=1}^{N} \sum_{q=1}^{Q} v^{i,q} B^{i,q}(x, y; t), \tag{26}$$

where

$$v^{i,q} = \frac{\langle F(x, y; t), B^{i,q}(x, y; t) \rangle}{\langle B^{i,q}(x, y; t), B^{i,q}(x, y; t) \rangle} = \frac{\int_0^{T_{\mathrm{tar}}} \iint_{\Omega_{\mathrm{tar}}} F_{\mathrm{tar}}(x, y; t) \left(B^{i,q}(x, y; t) \right)^* \mathrm{d}x\mathrm{d}y\mathrm{d}t}{\langle B^{i,q}(x, y; t), B^{i,q}(x, y; t) \rangle}. \tag{27}$$

In Supplementary Equation (26), $\mathbf{P}_{\mathrm{syn}}$ denotes the projection operator corresponding to $\mathcal{S}$, and $v^{i,q}$ can be computed efficiently with the use of FT.

Let $F_{\mathrm{syn}}(x, y; t) = F_{\mathrm{pr}}\,(x, y; t)$. Combining Supplementary Equation (21) and (26), we can obtain the spectrum of the excitation signals as

$$S^{i,q} = r^i \exp\left(\mathrm{j} k_0^q r^i\right) v^{i,q}. \tag{28}$$

Finally, by the Fourier transform in the time domain, the excitation signal of each antenna can be calculated as

$$S^i(t) = \Re\left[\sum_{q=1}^{Q} S^{i,q} \exp(\mathrm{j}2\pi f^q t) \right]. \tag{29}$$

Supplementary Note 2: Details on design of the spherical antenna array

We design a spherical array with 9×9 antennas, with the position $\boldsymbol{r}^i = (r^i, \theta^i, \varphi^i), (i = 1,2, \dots, N)$ of the i-th antenna. As derived in equation (8), the spatial-frequencies corresponding to the i-th antenna are

$$\begin{cases} k_x^{i,q} = -k_0^q \sin\theta^i \cos\varphi^i \\ k_y^{i,q} = -k_0^q \sin\theta^i \sin\varphi^i, \\ k_z^{i,q} = -k_0^q \cos\theta^i \end{cases} \tag{30}$$

where the superscript q corresponds to the q-th temporal frequency. Supplementary Equation (30) implies that the uniform sampling of k_x and k_y requires the antennas to be placed at equal $\sin\theta\cos\varphi$-interval and equal $\sin\theta\sin\varphi$-interval. The position (x^i, y^i, z^i) of the i-th antenna can be expressed as

$$\begin{cases} x^i = R\sin\theta^i \cos\varphi^i \\ y^i = R\sin\theta^i \sin\varphi^i, \\ z^i = R\cos\theta^i \end{cases} \tag{31}$$

where R denotes the radius of the array. Thus, we can derive that the antennas should be places at equal x-interval and equal y-interval. The detailed diagram of the array is shown in Supplementary Figure 1, where $R = 3.3m$, $\beta = 30°$, and $d = R\sin\beta/8 = 0.21\text{m}$ denotes the intervals along the x-direction and y-direction. The detailed geometries of the designed Vivaldi antenna are listed in the Supplementary Table 1.

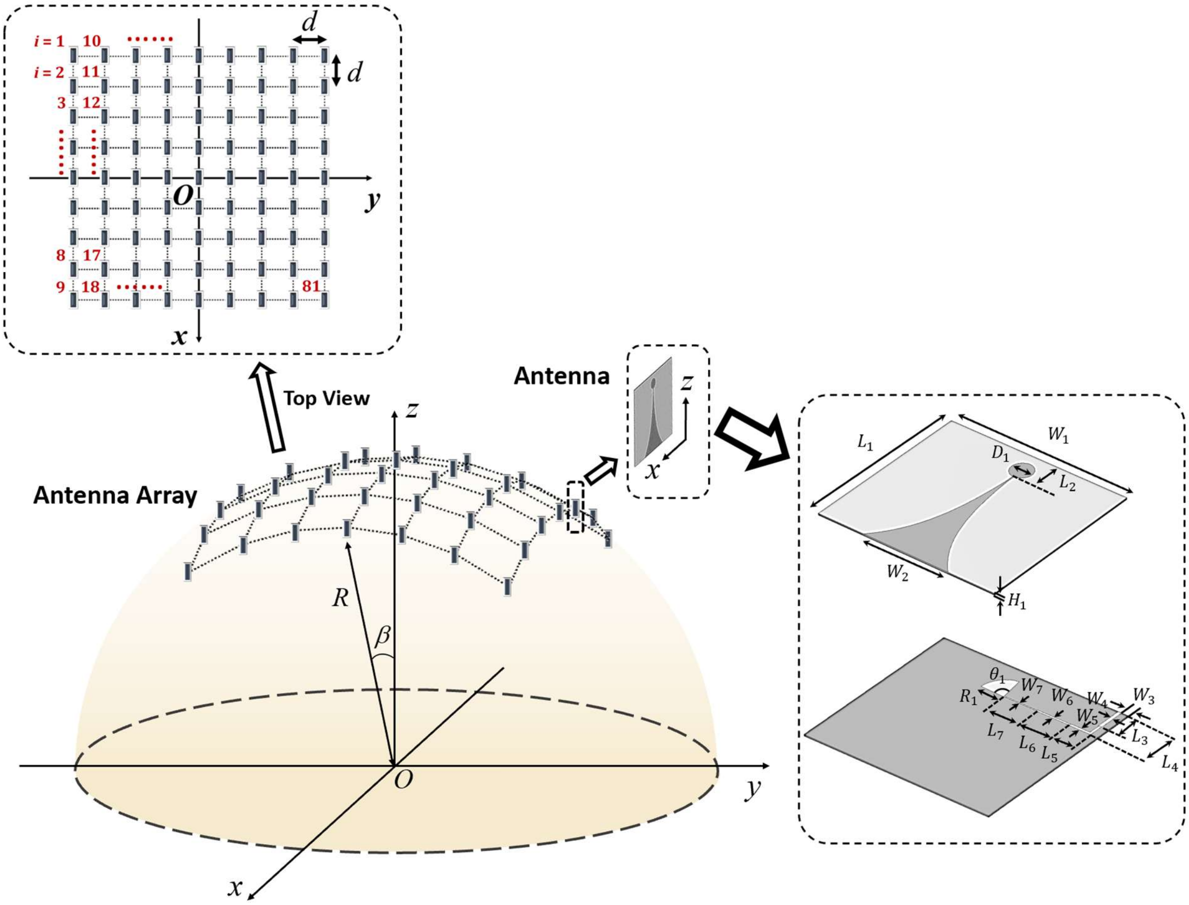

Supplementary Figure 1. Detailed diagram of the spherical antenna array and antenna.

Supplementary Table 1. Geometries of the designed Vivaldi antenna.

Dimension	Value	Dimension	Value
L_1	150mm	W_1	167.6mm
L_2	28.8mm	W_2	80.2mm
L_3	21.46mm	W_3	5.65mm
L_4	31.03mm	W_4	3mm
L_5	19.31mm	W_5	2.25mm
L_6	30mm	W_6	1.59mm
L_7	30mm	W_7	0.92mm
H_1	1.6mm	R_1	21.06mm
D_1	19.66mm	θ_1	80°

Supplementary Note 3: Details on the mean squared error calculation

In the simulation, the four-dimensional (4D) desired field #1 and the corresponding synthesized field are all sampled into 4D matrices with dimension (128, 128, 28, 201). The number of sample points along the *x*-, *y*- and *z*-directions is 128, and the number along time is 201. Reshaping them into 421527552-by-1 vectors, the mean squared error (MSE) between them is calculated by

$$MSE = \frac{\left\|\boldsymbol{F}_1 - \boldsymbol{F}_{\mathrm{syn}}\right\|^2}{128 \times 128 \times 128 \times 201} \times 100\% \,, \tag{32}$$

where $\boldsymbol{F}_1$ and $\boldsymbol{F}_{\mathrm{syn}}$ denote the vectors corresponding to the desired field #1 and synthesized field, respectively. $\|\cdot\|$ denotes the Euclidean norm.

Supplementary Note 4: The calculation time of PST-FT for 4D synthesis

The PST-FT methods is performed to solve 4D synthesis problems with a computer of an Intel(R) Xeon(R) Gold 6230 at 2.10GHz and 256GB RAM at 2933MHz. Supplementary Table 2 lists the calculation time under different array scales, field durations, and spatial sampling point, where the first row corresponds to the desired field #1 in the main text. It is evident that the growth rate of calculation time is much lower than that of the number of antennas, field duration, and field sampling points, demonstrating an obvious advantage in calculation time.

Supplementary Table 2. Calculation time of PST-FT for 4D synthesis

Antenna Array	Desired field Duration (ns)	Sampling points of 3D synthesis area	Calculation time
9×9	10	128×128×128	49 s
		160×160×160	1.4 minutes
	20	128×128×128	2.0 minutes
		160×160×160	6.5 minutes
13×13	10	128×128×128	1.5 minutes
17×17	10	128×128×128	2.1 minutes
21×21	10	128×128×128	2.5 minutes

Supplementary Note 5: Limitations on the synthesized field of the designed array

Owing to the discrete sampling of the spatio-temporal frequency spectrum, the synthesized field has the periodic prolongation in both temporal and spatial domains. The antennas are placed at equal intervals ($d = 0.21\text{m}$) along the x-and y-directions, as mentioned in Supplementary Note 2. At the temporal frequency of f^q, the sample intervals Δk^q for k_x and k_y are calculated by combining Supplementary Equation (30) and (31):

$$\Delta k^q = -k_0^q \cdot \frac{d}{R} = -\frac{2\pi d}{cR} f^q, \tag{33}$$

Subsequently, at the frequency of f^q, the period T_{syn}^q of the synthesized field along x- and y-directions can be obtained as

$$T_{syn}^q = \frac{2\pi}{\Delta k^q}. \tag{34}$$

The sample intervals and the period corresponding to the designed array are shown in the Supplementary Figure 2. Similarly, we obtain that the temporal period of the synthesized field is 40ns. Thus, to avoid the aliasing distortion in the synthesized field, the size must be limited to 2400mm and the duration must be limited to 40ns. Conversely, if the size of the desired field exceeds this limit, more intensive array is required. And the desired field with longer duration requires more intensive temporal frequency sampling.

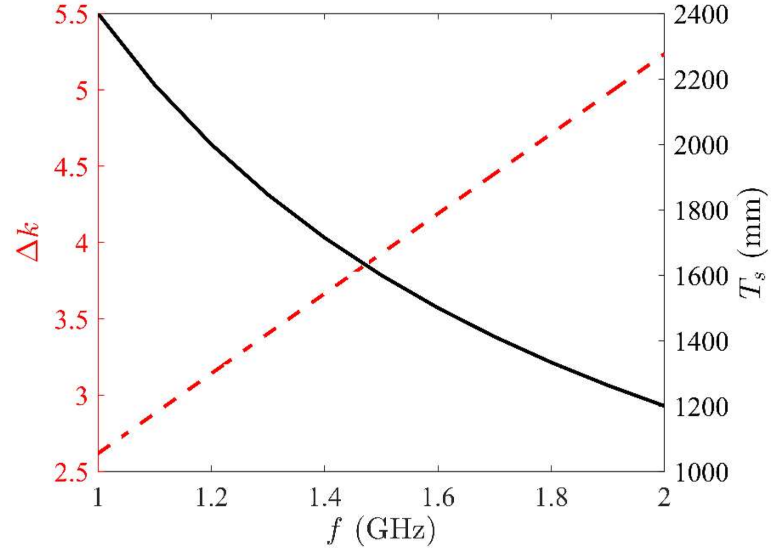

Supplementary Figure 2. The sample intervals and the period corresponding to the designed array at 1-2GHz. The sample intervals for k_x and k_y is shown by the red dashed line. The period of the synthesized field along *x*- and *y*-directions is shown by the black line.

Supplementary Note 6: Feasible method to depress the sidelobe levels

To depress the sidelobe levels, some optimization algorithms are required. Among them, the method based on smooth reference masks (SRMs) can quickly synthesizing the microwave fields of the desired sidelobe levels[1]. It builds the SRM by changing the width of the original desired field and spatial filtering. Then the synthesized field corresponding to the SRM will have lower sidelobe levels.

Since the presented PST-FT method synthesizes EM field from the perspective of spatio-temporal frequency domain, it is compatible with the spatial filtering method. We can combine these two theories to achieve the synthesis of low sidelobe time-varying fields.

For instance, let us consider a time-varying field focused at the position $(x, y) = (0, -500\text{mm})$ at 1ns. Applying projected space-time Fourier transform (PST-FT) theory, we first obtain the original synthesized field, as shown by the black line in Supplementary Figure 3. Then, by reducing the diameter of the expected focus from 450mm to 300mm and spatially-filtering its spatial spectrum at each temporal frequency, we obtain the SRM. The spatial filter function $\tilde{\varsigma}^q(k_x, k_y)$ at each temporal frequency of f^q is

$$\tilde{\varsigma}^q(k_x, k_y) = \exp\left(-\left(\frac{k_x}{0.49k_0^q}\right)^2 - \left(\frac{k_y}{0.49k_0^q}\right)^2\right) \tag{35}$$

with the full width at half maximum (FWHM) $0.81k_0^q$, where $k_0^q = 2\pi f^q/c$, c denotes the wave speed. Synthesizing this SRM via the PST-FT theory, we obtain the improved synthesized field, as shown by the red dashed line in Supplementary Figure 3. Compared with the original synthesized field, the sidelobe is reduced by about 10dB, while the focusing width is narrowed as a corresponding cost.

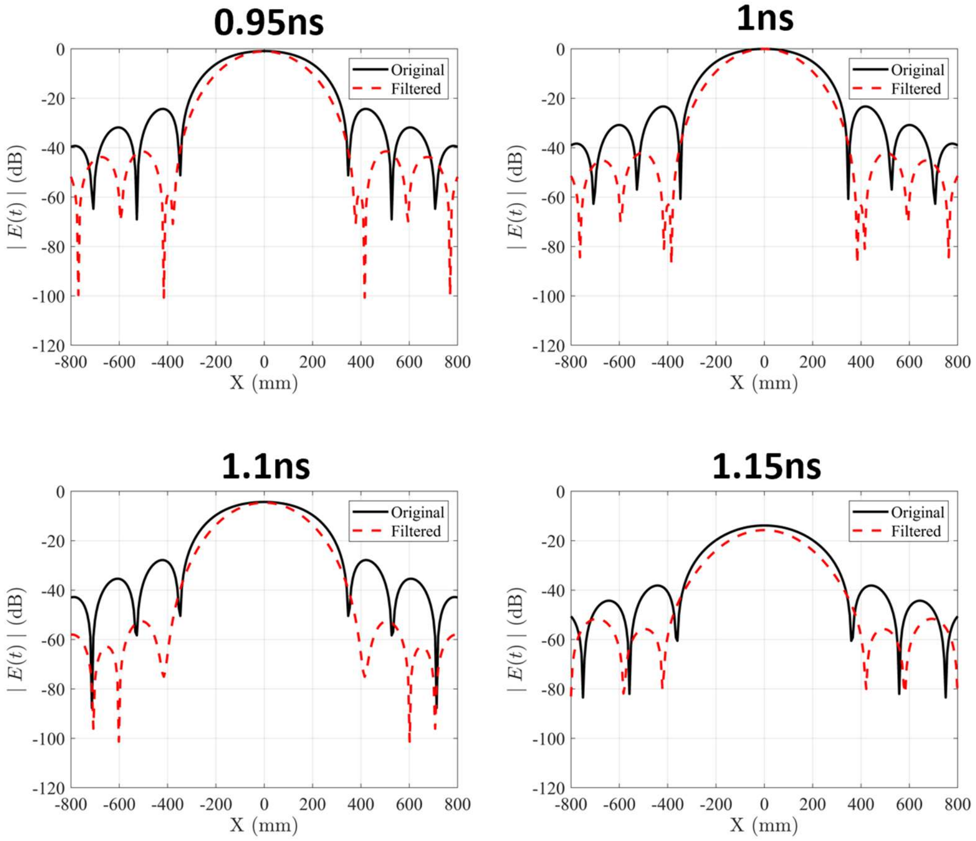

Supplementary Figure 3. The intensity profiles along the y = -500mm at different moments (0.95ns, 1ns, 1.1ns, and 1.15ns). The black line denotes the original synthesized field. The red dashed line denotes the improved synthesized field.

Supplementary Note 7: Comparison of the polarization components of the desired and synthesized electric fields

Supplementary Table 3 lists the maximum field intensity of different components of the normalized desired and synthesized electric fields in the simulation. Since the designed antenna is x-polarized and we neglect the components orthogonal to its polarization, all the desired fields are specified as x-polarized electric fields. In the rightmost column, the results show that y- and z-polarized components of the synthesized fields exhibit at least 10 dB suppression relative to the x-polarized. They indicate that under far-field conditions, the synthesized fields are predominantly contributed by the polarization component consistent with the excited antenna.

Supplementary Table 3. Maximum field intensity of different components of the normalized desired and synthesized fields

Corresponding desired field	Polarization component	Maximum intensity of the desired field (dB)	Maximum intensity of the synthesized field (dB)
#1	x	0	0
	y		-25.1
	z		-12.8
#2	x	0	0
	y		-25.5
	z		-14.4
#3	x	0	0
	y		-22.7
	z		-10.4

Supplementary Reference

1. Guo, S., Zhao, D. & Wang, B. -Z. Dimension-reduced optimization for uniform near-field synthesis of irregular arrays. *IEEE Antennas. Wireless Propag. Lett.* **21**, 908-912 (2022).
