# Supplementary material for: Continuous spatio-temporal synthesis of electromagnetic fields by projected space-time Fourier transform
Source: Commun Eng. 2025 Jun 17;4:110. doi: 10.1038/s44172-025-00448-9 (PMC12174354; doi:10.1038/s44172-025-00448-9)
Supplement: Supplementary file 2 — Description of Additional Supplementary Files [file 44172_2025_448_MOESM2_ESM.pdf]

# Description of Additional Supplementary Files

**File name: Supplementary Movie 1**

**Description:** Simulated Time-Varying Field (Ex-field intensity) of Desired Field #1.

**File name: Supplementary Movie 2**

**Description:** Simulated Time-Varying Field (Ex-field intensity) of Desired Field #2.

**File name: Supplementary Movie 3**

**Description:** Simulated Time-Varying Field (Ex-field intensity) of Desired Field #3

**File name: Supplementary Data 1**

**Description:** Excitation signals 1 and 2 (S1 and S2) shown in the Fig. 3 of the main text.

**File name: Supplementary Data 2**

**Description:** Excitation signals (Si, i = 1, 2, …, 81) of antenna array to synthesize the desired field #1.

**File name: Supplementary Data 3**

**Description:** Excitation signals (Si, i = 1, 2, …, 81) of antenna array to synthesize the desired field #2.

**File name: Supplementary Data 4**

**Description:** Excitation signals (Si, i = 1, 2, …, 81) of antenna array to synthesize the desired field #3.
